# The Lipid Droplet–Associated Hydrolase Is Associated With Obesity and Adipose Tissue Inflammation in Children

**DOI:** 10.1002/oby.70216

**Published:** 2026-05-28

**Authors:** Claudia Vales‐Villamarín, Antje Berthold, Martin Lacher, Antje Körner, Kathrin Landgraf

**Affiliations:** ^1^ Center for Pediatric Research, University Hospital for Children and Adolescents, Medical Faculty University of Leipzig Leipzig Germany; ^2^ Department of Pediatric Surgery University of Leipzig Leipzig Germany; ^3^ Helmholtz Institute for Metabolic Obesity and Vascular Research (HI‐MAG) of the Helmholtz Zentrum München at the University of Leipzig and University Hospital Leipzig Leipzig Germany; ^4^ LIFE–Leipzig Research Center for Civilization Diseases Medical Faculty, University of Leipzig Leipzig Germany; ^5^ German Center of Child and Adolescent Health (DZKJ) Leipzig/Dresden and Munich Partner Sites Germany

**Keywords:** adipocyte differentiation, adipose tissue inflammation, children, LDAH, obesity

## Abstract

**Objective:**

The lipid droplet–associated hydrolase (LDAH) is a lipid droplet–associated protein with an uncharacterized role in human adipose tissue (AT) and obesity; we aimed to investigate its role in human AT and its relevance for childhood obesity.

**Methods:**

*LDAH* variant rs13385191 and gene expression were analyzed in a cross‐sectional study of subcutaneous AT samples from 296 children (120 girls, 176 boys; ages 0–18; BMI SDS −2.7 to 4.3), and an association with obesity and AT biology was studied. An effect of *LDAH* on adipogenesis was investigated in SGBS preadipocytes.

**Results:**

Minor allele carriers of rs13385191 showed lower AT *LDAH* expression compared to non‐carriers (*p* < 0.001) and a trend toward higher BMI SDS, which was, however, not statistically significant (*p* = 0.062). Consistently, study participants with lower *LDAH* expression showed higher BMI SDS (*p* = 0.005). A negative correlation was found between *LDAH* and macrophage infiltration into AT after controlling for age (*R* = −0.132; *p* = 0.039), and high *LDAH* expression was associated with lower circulating TNFα (*p* = 0.017). *LDAH* expression increased during SGBS adipocyte differentiation, while its knockdown did not alter differentiation. In line with results from AT, TNFα significantly reduced *LDAH* expression in SGBS cells (*p* = 0.009).

**Conclusions:**

LDAH seems to have a role in AT inflammation and the development of obesity in children.

## Study Importance

**What is already known?:**

- The LDAH protein localizes to lipid droplets, contains a lipase/hydrolase domain with low enzymatic activity in mammals, and has been linked to prostate cancer and lipid homeostasis in model systems.
- A role of LDAH in adipose tissue of humans in general and children in particular, as well as a potential relevance for obesity, has not been investigated so far.

**What does this study add?:**

- This is the first study in humans showing associations between genetic *LDAH* variants, *LDAH* gene expression in adipose tissue, BMI SDS, and particular adipose tissue inflammation in children.
- The association between *LDAH* expression and adipose tissue inflammation was confirmed by characterizing *LDAH* in human adipocytes in vitro.

**How might these results change the direction of research or the focus of clinical practice?:**

- The findings suggest that LDAH might serve as a novel biomarker or therapeutic target for childhood obesity and adipose tissue dysfunction associated with metabolic disease, which should be addressed in future research.

## 1 Introduction

Overweight and obesity rates continue to increase, making obesity one of the most prevalent pathologies worldwide [1]. Recent surveillance data suggest that close to one in four European children aged 7–9 years suffer from overweight or obesity [2]. Since childhood obesity is likely to persist into adulthood and early signs of adipose tissue (AT) dysfunction and metabolic disease are already evident in children [3, 4], addressing this pathology from an early age is of great importance, particularly because excess adiposity promotes insulin resistance and increases the risk of obesity‐related comorbidities, including youth‐onset type 2 diabetes (T2D), which has shown a clear upward trend in Europe over the last decade [5]. Consistently, a large meta‐analysis in children and adolescents reported markedly higher prevalence of cardiometabolic comorbidities in obesity, including dysglycemia/prediabetes (1.4‐fold), elevated blood pressure (4.4‐fold), and NAFLD (26.1‐fold) [6].

The excess of energy resulting from a positive energy balance is stored in the form of lipids within the body. Specifically, white adipose tissue (WAT), primarily composed of adipocytes, acts as the main storage of fat in our body [7]. In recent years, studies have highlighted the increase in adipocyte size as the main cause of the unhealthy expansion of AT, leading to its dysfunction and subsequently the development of comorbidities such as T2D and cardiovascular disease [3, 8].

White adipocytes are characterized by a high content of lipid droplets (LD), which serve as a lipid deposit within the cells [9]. The lipid droplet contains several associated proteins (lipid droplet‐associated proteins, LDAPs) [9]. Among them are members of the perilipin family, such as PLIN1, associated with severe familial partial lipodystrophy [10, 11], and the CIDE family, such as CIDEA and CIDEC that, like PLIN1, have been associated with obesity and T2D [12].

The lipid droplet–associated hydrolase (LDAH) is a LDAP that is highly expressed in tissues with high lipid content such as liver or AT [13]. However, its direct role in AT and during adipocyte formation and its relevance for obesity and metabolic disease remain unclear. Contradictory results have been found regarding its possible role in lipid accumulation and metabolism [14, 15, 16]. Studies in cell models indicate that LDAH can limit ATGL‐driven lipolysis and favor TAG storage [15, 17]. LDAH is also expressed in macrophages, where it is linked to cholesterol ester turnover and efflux [13]. In vivo studies in an atherosclerosis context further support a role for LDAH in mobilizing esterified sterols and shaping macrophage programs toward a less inflammatory phenotype [18]. In adipocyte models, LDAH increases during 3 T3‐L1 differentiation and is upregulated in expanding mouse WAT under Western diet [15]. However, studies in knockout mice show mixed results and did not find evidence for a major function in triglycerol metabolism [14, 16].

Previous studies have also examined *LDAH* gene variants in humans. Although *LDAH* has been mainly highlighted in genetic studies of prostate cancer [14, 16, 19, 20, 21, 22, 23, 24, 25, 26, 27, 28], the same locus has also been linked to cardiometabolic traits [14]. Variants within the *LDAH* linkage disequilibrium region have been reported to be associated with lipid‐related traits and anthropometric measures [14], and they have been associated with both T2D and pulse pressure [28, 29].

The close association of LDAH with lipid droplets, together with high expression in AT, makes it highly relevant for investigating a potential role of LDAH in adipocyte differentiation and in the development of obesity and related comorbidities. The absence of confounding factors, such as smoking or alcohol consumption, makes the pediatric population highly valuable for studying the etiology and pathogenesis of obesity. Importantly, childhood obesity is already associated with measurable obesity‐related molecular and cellular remodeling, providing an opportunity to investigate early pathogenic mechanisms before overt clinical complications develop [3].

Therefore, in this study, we hypothesized that LDAH might play a role in processes related to AT biology and function during the development of obesity in children. We aimed to analyze the association of LDAH with obesity and clinical comorbidities in a population of children and its potential involvement in the differentiation of human adipocytes in vitro.

## 2 Methods

### 2.1 Participants and Samples (Leipzig Adipose Tissue Childhood Cohort)

Subcutaneous AT samples were collected from 296 European children (ages 0–18, *n* = 292 with information on body mass index standard deviation score [BMI SDS] available), with samples taken from orthopedic surgery (knee, hip, arm, shoulder, leg, foot, coccyx, *n* = 187), herniotomy or orchidopexy (inguinal area, *n* = 84), abdominal surgery (*n* = 12), or other surgeries (*n* = 13). Exclusion criteria included diabetes, generalized inflammation, malignant diseases, genetic syndromes, and permanent immobility. Written informed consent was obtained from all parents. The study was approved by the local ethics committee (Ethics Committee of the Medical Faculty) (265–08, 265–08‐ff; ClinicalTrials.gov NCT02208141).

BMI is given as BMI SDS standardized to age‐ and sex‐specific German reference data with a cutoff of 1.28 and 1.88 SDS defining overweight and obesity in children, respectively [30]. Puberty stage is given as Tanner stage determined from assessment of pubic hair (PH) growth with PH1 representing pre‐puberty, PH2‐4 representing puberty, and PH5‐6 representing post‐puberty. Fasting blood samples were obtained prior to surgery. Levels of leptin, high‐sensitivity C‐reactive protein (hs‐CRP), TNFα, cholesterol, glucose, and insulin were measured by a certified laboratory. Homeostasis model assessment—insulin resistance (HOMIR) was calculated to evaluate insulin resistance [31].

Assessment of AT function was performed using established protocols described in detail by Landgraf et al. [3]. Briefly, adipocytes and stromal vascular fraction (SVF) cells were isolated by collagenase digestion. Adipocyte diameter and number were determined using a Coulter counter (Multisizer III, Beckmann Coulter). Macrophage infiltration was analyzed by immunostaining of AT sections with CD68 antibody (M0718, DAKO). Basal and isoproterenol‐stimulated lipolytic activity of adipocytes was assessed using Free Glycerol Reagent (Sigma) and is given as glycerol release in ng/mL per 1000 adipocytes. Doubling time and adipocyte differentiation capacity of SVF cells were analyzed as previously described [32].

### 2.2 SNP Genotyping

Genomic DNA was obtained from blood samples. The SNP rs13385191 in *LDAH* was selected and genotyped using a predesigned TaqMan SNP Genotyping Assay (C___3227785_10, Applied Biosystems) on a QuantStudio 3 Real‐Time PCR System (Applied Biosystems). Quantitative real‐time PCR (qPCR) was performed with a mixture of 10 ng of genomic DNA, TaqMan SNP Genotyping Assay (20X), and TaqMan Genotyping Master Mix (Applied Biosystems). Samples were cycled under the recommended conditions: 95°C for 10 min, 95°C for 15 s, and 60°C for 1 min, repeated over 40 cycles.

### 2.3 Cell Line and Culture Conditions

Human Simpson–Golabi–Behmel syndrome (SGBS) [33] cells were kindly provided by Martin Wabitsch (University of Ulm). Cells were cultured at 37°C with 5% CO_2_ in basal medium consisting of DMEM/Ham F12 medium (11330–032, Life Technologies) supplemented with 10% fetal bovine serum (S0615, Merck), 33‐mmol/L biotin (B4639, Sigma), and 17‐mmol/L pantothenic acid (P5155, Sigma).

### 2.4 In Vitro Adipocyte Differentiation

Differentiation of SGBS cells was induced by treating confluent cells with serum‐free medium containing 20nM insulin (I‐2643, Sigma), 100nM hydrocortisone (H0888, Sigma), 0.2nM triiodothyronine (T6397, Sigma), and 0.13nM apo‐transferrin (T1147, Sigma). During the first 4 days, the medium was further supplemented with 25nM dexamethasone (D1756, Sigma), 500μM isobutyl‐1‐methylxanthine (I‐5879, Sigma), and 2μM rosiglitazone (R2408, Sigma). Samples were collected on the day of induction (d0) and on Days 2, 4, 6, and 8 after induction of adipogenesis for expression and lipid staining analysis.

### 2.5 siRNA‐Mediated Knockdown

Small interfering RNA (siRNA) transfections of SGBS cells were performed using the Neon Transfection System 100 mL Kit (Invitrogen). Electroporation was optimized to pulse voltage 1300 V, pulse width 20 ms, pulse number 2, and a cell density of 6 × 10^6^ cells/mL [34]. LDAH‐specific ON‐TARGETplus SMARTpool siRNA (L‐009926‐01‐0005) and ON‐TARGETplus control reagents (D‐001810‐10‐05, Dharmacon) were used at a final concentration of 500 nM. After electroporation, 150,000 cells per well were seeded in 12‐well format and, after 1 day, differentiated into mature adipocytes. Knockdown efficiency was confirmed using qPCR.

### 2.6 Differentiation Capacity

Adipocytes at Day 8 post induction were fixed in Roti‐Histofix 4% (Carl Roth GmbH) and double‐stained with Nile Red (19123, Sigma) and Hoechst 33342 (B2261, Sigma). Differentiation capacity of SGBS cells is represented as percent Nile Red/Hoechst double‐stained cells from the total number of Hoechst‐positive cells per well, determined from microscopic images (EVOS, Fisher Scientific).

After Nile Red/Hoechst staining, Oil Red O staining was performed in adipocytes at Day 8 post induction in a representative experiment. Cells were stained with Oil Red O solution (0.3% in 60% isopropanol; O0625, Sigma) for 15 min. After incubation, the plates were washed in distilled water and Oil Red O was extracted with isopropanol. Absorbance was determined at 540 nm using the FLUOstar OPTIMA (BMG LABTECH).

### 2.7 TNFα Treatment of Differentiated SGBS Cells

SGBS preadipocytes were cultured and differentiated as previously described [35]. At Day 8 of differentiation, SGBS cells were stimulated with 10 ng/mL of recombinant human TNFα (210‐TA, R&D Systems) in differentiation medium for 6 h and 24 h before harvesting cells for RNA isolation and qPCR analyses.

### 2.8 RNA Isolation and qPCR Analysis

Total RNA was extracted from SGBS cells using RNeasy Mini Kit (QIAGEN), including on‐column DNA digestion according to the manufacturer's instructions. Isolated RNA from AT samples was obtained as previously described [34]. Reverse transcription of 500 ng of RNA was performed using M‐MLV Reverse Transcriptase (Invitrogen) and random hexamer primers (Promega).

qPCR was performed as previously described [34]. In both AT samples and SGBS cells, absolute copy number for each gene in each sample was calculated from a standard curve. Housekeeping (HK) genes (*TBP* [TATA‐box binding protein], *ACTB* [β‐actin], and *HPRT* [hypoxanthine‐guanine phosphoribosyl‐transferase] for AT samples; *TBP* and *ACTB* for SGBS cells) were first scaled gene‐wise, averaged across technical replicates, and combined into a sample‐specific HK factor (mean of scaled HKs) used for normalization of target gene expression. Primer and probe sequences of housekeeping genes used for qPCR are listed in Table 1.

**TABLE 1:** Sequence of primers and probes used for quantitative real‐time PCR (qPCR).

Symbol	Gene name	Forward primer	Reverse primer	Probe
*LDAH*	Lipid droplet‐associated hydrolase	Predesigned TaqMan assay (Hs01092034_m1, Applied Biosystems)		
*PPARG*	Peroxisome proliferator‐activated receptor gamma	GATCCAGTGGTTGCAGATTACAA	GAGGGAGTTGGAAGGCTCTTC	TGACCTGAAACTTCAAGAGTACCAAAGTGCAA
*ACTB*	β‐Actin	TGAGCGCGGCTAC AGCTT	CCTTAATGTCACG CACGATTT	ACCACCACGGCCGAGCGG
*TBP*	TATA‐box‐binding protein	TTGTAAACTTGAC CTAAGACCATTGC	TTCGTGGCTCTCTT ATCCTCATG	AACGCCGAATATAATCCCAAGCGGTTTG
*HPRT*	Hypoxanthine‐guanine phosphoribosyl‐transferase	GGCAGTATAATCC AAAGATGGTCAA	GTCTGGCTTATAT CCAACACTTCGT	CAAGCTTGCTGGTGAAAAGGACCCC

*Note*: Primers and probes are given in 5′‐3′ direction. Probes were labeled with the reporter 5′‐FAM or 5′‐HEX for TBP and the quencher 3′‐TAMRA.

### 2.9 Statistical Analysis

Statistical analyses of cell culture experiments and graphical representations were performed using Prism 10 (GraphPad Software). Statistical analysis of human AT samples and genotyping were performed using Statistica 10 (StatSoft) and IBM SPSS Statistics 26.0 (SPSS Inc.). Data in bar plots are presented as mean ± SEM and variables were log‐transformed when needed to approximate normality.

Group comparisons were performed using Student's *t*‐test/ANOVA or Mann–Whitney *U* test, as appropriate; categorical variables were compared using chi‐square or Fisher's exact test. Associations were assessed using Pearson and partial correlations, and potential confounders were addressed using univariate GLM models with relevant covariates. A *p* value of < 0.05 was considered statistically significant.

## 3 Results

### 3.1 The Variant rs13385191 in *LDAH* Is Associated With *LDAH* Expression in Human AT

**FIGURE 1:**
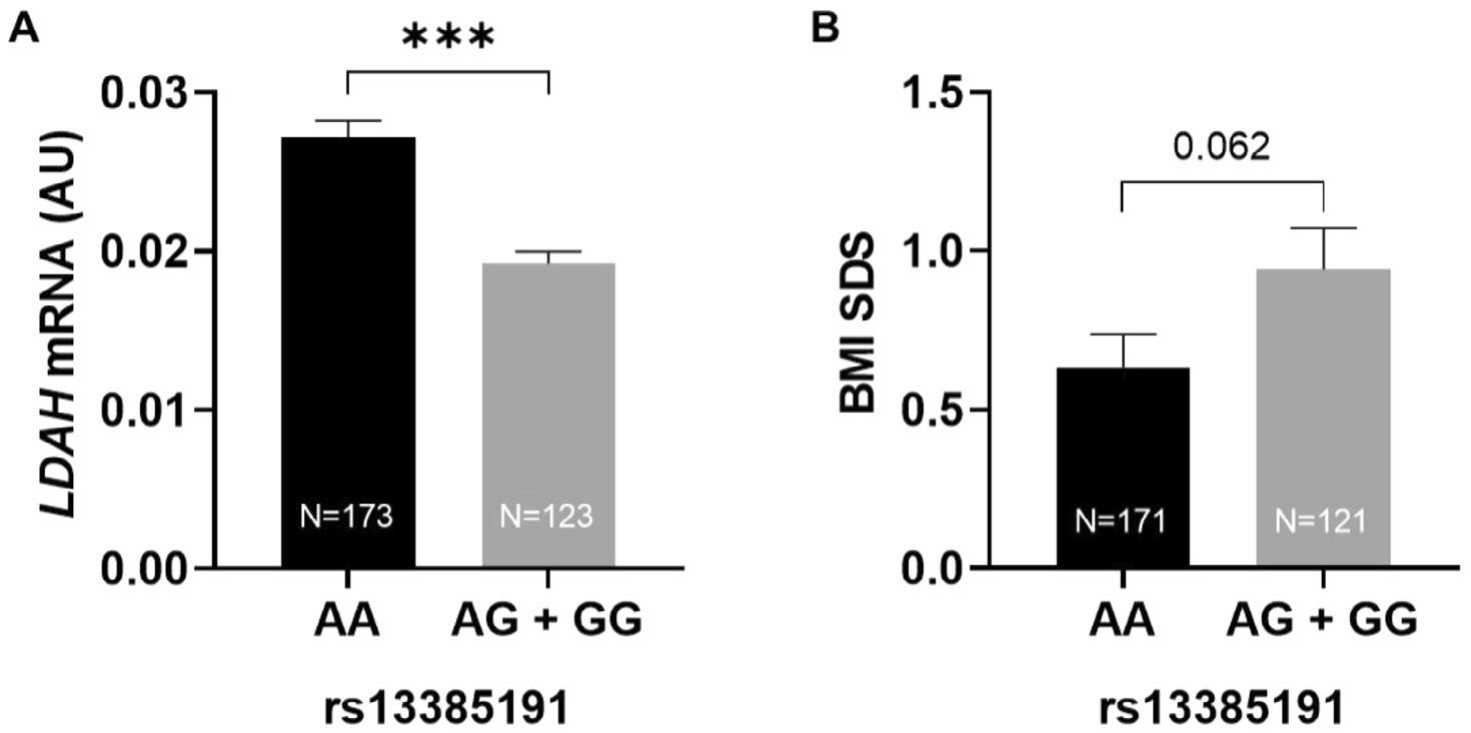
The rs13385191 SNP in *LDAH* is associated with *LDAH* mRNA expression in AT from children. (A) *LDAH* mRNA expression levels in AT stratified for the rs13385191 genotype in a dominant model. (B) BMI SDS according to rs13385191 genotype in a dominant model. Data in bar plots indicate mean ± SEM. AT, adipose tissue; LDAH, lipid droplet‐associated hydrolase. ***p < 0.001.

First, we aimed to evaluate the association of genetic variants in *LDAH* with childhood obesity and *LDAH* expression in AT of children. For this, we selected and genotyped the *LDAH* variant rs13385191, which had previously been shown to be associated with *LDAH* expression in other tissues, in DNA samples of our Leipzig Adipose Tissue Childhood Cohort (*N* = 296) [22, 25, 27]. Genotyping the variant showed no significant deviation from Hardy–Weinberg equilibrium (*p* = 0.099, Table 2). Carriers of the minor G allele showed significantly lower AT *LDAH* mRNA expression than AA participants (*p* < 0.001, Figure 1A). Furthermore, carriers of the G allele showed a tendency towards a higher BMI compared to AA participants (Figure 1B), although this was not significant (*p* = 0.062). Our observations indicate that the *LDAH* variant rs13385191 affects *LDAH* expression levels in AT and a potential association with obesity in children.

**TABLE 2:** Allelic and genotypic frequencies of rs13385191G in DNA samples of the Leipzig Adipose Tissue Childhood Cohort (*N* = 296).

rs13385191G	*N*	%
AA	173	58.4
AG	113	38.2
GG	10	3.4
A	459	77.5
G	133	22.5

### 3.2 *LDAH* mRNA Expression in AT Is Associated With Obesity in Children

**FIGURE 2:**
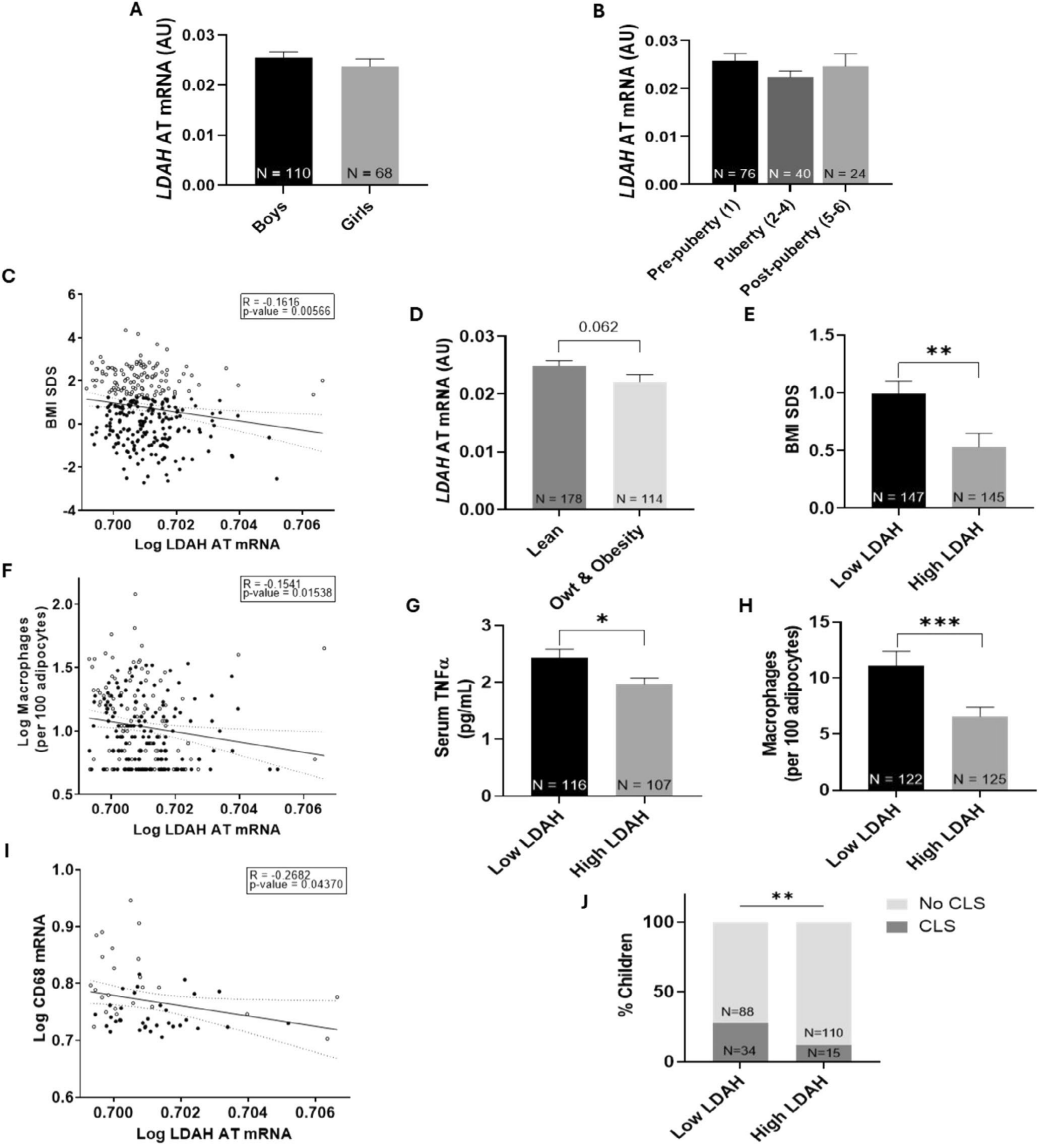
*LDAH* mRNA expression in AT of children is associated with obesity and AT inflammation. (A) *LDAH* mRNA expression in AT by sex, only in lean children. (B) *LDAH* mRNA expression in AT stratified by pubertal stage, only in the lean subgroup. Tanner stages (PH) are indicated in brackets. (C) Scatterplot showing association of *LDAH* mRNA expression in AT and BMI SDS. (D) *LDAH* mRNA expression in AT stratified by weight category (lean vs. overweight and obesity) of children. (E) Mean BMI SDS of children with Low vs. High *LDAH* mRNA expression in AT (stratified by median). (F) Scatterplot showing association of *LDAH* mRNA expression in AT and macrophages per 100 adipocytes. (G) Serum levels of TNFα in children with Low vs. High *LDAH* mRNA expression in AT (stratified by median). (H) Macrophage infiltration in AT of children with Low vs. High *LDAH* AT mRNA expression (stratified by median). (I) Scatterplot showing association of *LDAH* mRNA expression in AT and *CD68* mRNA expression in SVF. (J) Percentage of AT samples with or without CLS in relation to *LDAH* mRNA expression level (Low LDAH vs. High LDAH, stratified by median), with statistical significance determined by chi‐square test. Data in bar plots indicate mean ± SEM of indicated samples sizes and statistical significance was analyzed using Student's *t*‐test/ANOVA. In scatterplots each dot represents one individual with lean children in black and children with overweight/obesity in white. The solid line represents the linear regression fit. Pearson's *r* and two‐sided *p* value are indicated. AT, adipose tissue; CLS, crown‐like structures; LDAH, lipid droplet‐associated hydrolase; PH, pubic hair; owt, overweight; TNFα, tumor necrosis factor α. *p < 0.05; **p < 0.01; ***p < 0.001.

To determine whether LDAH is associated with AT development, obesity, and parameters of AT function, we analyzed AT samples from children. Information about anthropometric and AT‐related parameters from children of the Leipzig Adipose Tissue Childhood Cohort is described in Table 3, showing significant signs of AT dysfunction and early metabolic alterations in the subgroup of children with overweight and obesity compared to lean children that were independent from age and sex (Table 3). We first aimed to investigate an association of *LDAH* expression in AT with normal development and sex of children in the subcohort of healthy lean children (*N* = 178). No significant correlations were found between *LDAH* mRNA expression and height SDS (*R* = −0.051; *p* = 0.519), sex (*R* = −0.071; *p* = 0.345), or puberty stage (*R* = −0.072; *p* = 0.400) in correlation analyses. Likewise, there were no significant differences in *LDAH* mRNA expression between sexes (Figure 2A) and pubertal stages (Figure 2B) in group comparison. However, age was shown to be significantly negatively correlated with AT *LDAH* mRNA expression in lean children (*R* = −0.175; *p* = 0.020).

**TABLE 3:** Characteristics of lean children and children with overweight and obesity included in the Leipzig Adipose Tissue Childhood Cohort.

	Lean			Overweight and obesity				
	N	Mean ± SEM	Range	N	Mean ± SEM	Range	*P1*	*P2*
Anthropometric and clinical parameters								
Boys/Girls (% Boys)	110/68 (61.8)			62/52 (54.4)			0.128	—
Age, years	178	8.5 ± 0.4	0.1–18.4	114	12.1 ± 0.4	0.3–18.0	**< 0.001**	—
Pubertal stage, PH	140	2.3 ± 0.1	1–6	96	3.1 ± 0.2	1–6	**< 0.001**	0.731
BMI SDS	178	‐0.1 ± 0.1	−2.7‐1.2	114	2.2 ± 0.1	1.3–4.3	**< 0.001**	**< 0.001**
Height SDS	163	0.0 ± 0.1	−3.3‐2.9	99	0.5 ± 0.1	−3.2‐2.7	**0.001**	**0.003**
Waist, cm^a^	123	62.2 ± 1.1	36.0–101.0	89	88.8 ± 1.7	50.0–154.0	**< 0.001**	**< 0.001**
Triceps skinfold thickness, mm^a^	97	15.8 ± 0.7	5.0–61.0	79	26.2 ± 0.9	10.2–40.0	**< 0.001**	**< 0.001**
Subscapular skinfold thickness, mm^a^	92	10.2 ± 0.5	4.0–25.8	78	23.6 ± 1.0	7.2–43.0	**< 0.001**	**< 0.001**
SBP, mmHg	85	109.8 ± 1.2	86.0–143.0	70	121.3 ± 1.7	82.0–156.0	**< 0.001**	**< 0.001**
DBP, mmHg	85	69.6 ± 1.0	46.0–95.0	70	73.7 ± 1.2	46.0–99‐0	**0.009**	0.092
AT parameters								
Adipocyte diameter	31	113.9 ± 2.6	1.9–2.1	42	124.1 ± 2.5	2.0–2.2	**0.006**	**0.024**
Proliferation and differentiation capacity of cells from the SVF								
Doubling time of cells from the SVF, h^a^	23	71.8 ± 11.9	18.9–205.0	23	64.9 ± 7.3	31.7–180.7	0.623	0.605
Differentiated cells (%)	20	30.1 ± 3.9	58.2–54.2	23	26.7 ± 3.9	0.2–74.0	0.537	0.759
Macrophage infiltration								
Macrophages per 100 adipocytes^a^	149	6.6 ± 0.6	0–29	97	12.3 ± 1.7	0–115	**0.003**	**0.032**
CD68 mRNA^a^	32	0.6 ± 0.1	0.1–1.6	24	1.4 ± 0.2	0.0–3.8	**< 0.001**	**< 0.001**
Number of children with CLS (%)	13 (8.7)			36 (37.1)			**< 0.001**	—
Metabolic function of adipocytes								
Basal lipolysis	15	0.5 ± 0.0	0.2–0.7	17	0.3 ± 0.0	0.0–0.7	**0.015**	0.054
Isoproterenol‐stimulated lipolysis^a^	15	2.1 ± 0.2	0.6–3.8	18	2.1 ± 0.3	0.4–5.1	0.981	0.753
Serum parameters								
Leptin, ng/mL^a^	98	6.1 ± 0.6	0.1–27.3	99	28.7 ± 2.3	0.6–99.0	**< 0.001**	**< 0.001**
hs‐CRP, mg/L^a^	130	0.8 ± 0.1	0.2–10.0	102	2.1 ± 0.3	0.2–18.4	**< 0.001**	**< 0.001**
TNFα, pg/mL^a^	125	2.3 ± 0.1	0.6–9.9	96	2.1 ± 0.2	0.6–9.5	0.433	0.732
Total cholesterol, mmol/L^a^	132	3.8 ± 0.1	2.0–8.8	102	3.8 ± 0.1	2.3–6.2	0.874	0.910
Glucose, mmol/L	132	4.6 ± 0.1	2.5–6.1	103	4.7 ± 0.1	3.1–7.0	0.064	0.073
Insulin, pmol/L^a^	132	42.8 ± 2.9	1.8–159.6	98	108 ± 7.0	5.0–359.7	**< 0.001**	**< 0.001**
HOMA‐IR^a^	131	−0.1 ± 0.0	0.0–5.6	98	0.4 ± 0.0	0.1–12.7	**< 0.001**	**< 0.001**
AT mRNA expression								
*LDAH* (AU)^a^	178	0.025 ± 0.001	0.004–0.072	114	0.022 ± 0.001	0.002–0.089	0.062	0.186

*Note*: The characteristics of the population, grouped by weight category, are expressed as mean ± SEM. Statistical significance for differences between groups was determined by Student's *t‐*test. For sex and occurrence of CLS, Chi‐square was used to analyze statistical significance. Adjustment for potential confounders was performed using a univariate general linear model (GLM). *P1*: *p* value from not adjusted analysis; *P2*: *p* values from analysis adjusting for sex and age. Significant *p* values are indicated in bold. Basal and isoproterenol‐stimulated lipolysis are given as glycerol release in (ng/mL)/1000 adipocytes.

Abbreviations: AT, adipose tissue; DBP, diastolic blood pressure; hs‐CRP, high‐sensitivity C‐reactive protein; PH, pubic hair; SBP, systolic blood pressure; SVF, stromal vascular fraction; TNFα, tumor necrosis factor α.

^a^ Statistical analyses were performed for log‐transformed parameters.

We next aimed to analyze a potential association of AT *LDAH* mRNA expression with obesity and AT dysfunction in the whole cohort including lean children and children with overweight and obesity. We found a significant negative correlation between *LDAH* expression in AT with BMI SDS, which persisted after controlling for sex and age (Figure 2C, Table 4). Although not significant, a tendency could also be observed in groups stratified for BMI category, with lower mRNA expression levels in participants with overweight or obesity compared to lean participants (Figure 2F). In line with this, after classifying children into two groups according to median *LDAH* mRNA expression, we observed a significantly lower mean BMI SDS in the High LDAH group compared to the Low LDAH group (Figure 2G). These findings suggest that AT *LDAH* mRNA expression levels are diminished in childhood obesity.

**TABLE 4:** Correlation analyses of AT LDAH mRNA expression with obesity‐related AT and serum parameters in children of the Leipzig Adipose Tissue Childhood Cohort.

	Model 1		Model 2		Model 3		
	*R*	*P*	*R*	*P*	*R*	*P*	*N*
Anthropometric parameters							
Age, years	**−0.121**	**0.037**	—	—	—	—	296
BMI SDS	**−0.162**	**0.006**	**−0.124**	**0.035**	—	—	292
Height SDS	−0.053	0.390	−0.049	0.436	−0.035	0.574	262
Waist, cm^a^	−0.085	0.218	−0.003	0.970	**0.144**	**0.037**	212
Triceps skinfold thickness, mm^a^	0.050	0.943	0.057	0.454	**0.190**	**0.012**	176
Subscapular skinfold thickness, mm^a^	−0.073	0.343	−0.026	0.735	0.114	0.143	170
SBP, mmHg	−0.036	0.656	0.008	0.918	0.055	0.501	155
DBP, mmHg	−0.081	0.319	−0.049	0.551	−0.021	0.798	155
AT parameters							
Adipocyte diameter	0.045	0.709	0.091	0.449	0.156	0.196	73
Proliferation and differentiation capacity of cells from the SVF							
Doubling time of cells from the SVF, h^a^	0.034	0.820	0.080	0.605	0.055	0.726	46
Differentiated cells (%)	−0.058	0.714	−0.078	0.627	−0.067	0.679	43
Macrophage infiltration							
Macrophages per 100 adipocytes^a^	**−0.154**	**0.015**	**−0.132**	**0.039**	−0.115	0.074	247
CD68 mRNA^a^	**−0.268**	**0.044**	−0.253	0.062	−0.224	0.107	57
Metabolic function of adipocytes							
Basal lipolysis	0.039	0.831	0.012	0.950	−0.064	0.742	32
Isoproterenol‐stimulated lipolysis^a^	−0.166	0.357	−0.156	0.403	−0.170	0.370	33
Serum parameters							
Leptin, ng/mL^a^	−0.127	0.076	−0.072	0.313	0.018	0.803	198
hs‐CRP, mg/L^a^	0.011	0.872	0.036	0.580	0.104	0.116	236
TNFα, pg/mL^a^	−0.058	0.390	−0.102	0.130	−0.092	0.174	223
Total cholesterol, mmol/L^a^	0.036	0.577	0.035	0.596	0.031	0.641	238
Glucose, mmol/L	−0.038	0.562	−0.036	0.585	−0.018	0.790	239
Insulin, pmol/L^a^	−0.050	0.449	0.030	0.648	0.096	0.148	234
HOMA‐IR^a^	−0.061	0.354	0.012	0.861	0.076	0.255	233

*Note*: Correlation coefficients (*R*) and *p* values (*P*) are presented. Significant correlations are indicated in bold. Model 1: Non‐adjusted; Model 2: Adjusted for sex and age; Model 3: Adjusted for sex, age, and BMI SDS.

Abbreviations: AT, adipose tissue; DBP, diastolic blood pressure; hs‐CRP, high‐sensitivity C‐reactive protein; SBP, systolic blood pressure; SVF, stromal vascular fraction; TNFα, tumor necrosis factor α.

^a^ Statistical analyses were performed for log‐transformed parameters.

### 3.3 AT Inflammation Is Associated With Lower *LDAH* AT mRNA Expression

Childhood obesity is associated with alterations in AT biology and function, and especially AT inflammation has been linked to the formation of obesity‐associated metabolic alterations [3].

We did not observe significant associations of *LDAH* expression with proliferation and differentiation capacity of SVF cells, metabolic function of adipocytes, adipokines, inflammatory serum parameters (e.g., leptin, hs‐CRP, TNFα), or HOMA‐IR (Table 4).

However, a significant negative correlation was found between AT *LDAH* mRNA expression and AT inflammation, as determined by the number of macrophages in AT, which remained significant after controlling for sex and age but was lost after adjusting for BMI SDS (Table 4, Figure 2F). These findings were consistent when adjusting for puberty stage instead of age (data not shown).

In line with the association with AT inflammation, the Low LDAH group showed significantly higher levels of serum TNFα than the High LDAH group (Figure 2G), as well as significantly more AT macrophages (Figure 2H), and AT *LDAH* mRNA expression was inversely correlated to *CD68* mRNA expression in the SVF (Table 4, Figure 2I). Furthermore, the presence of crown‐like structures (CLS) was measured and the population was divided between children with and without CLS, showing a higher percentage of children with CLS in the Low LDAH group compared to the High LDAH group (27.9% vs. 12.0%, respectively; Figure 2J).

### 3.4 *LDAH* Expression Is Upregulated During Adipocyte Differentiation in Vitro

**FIGURE 3:**
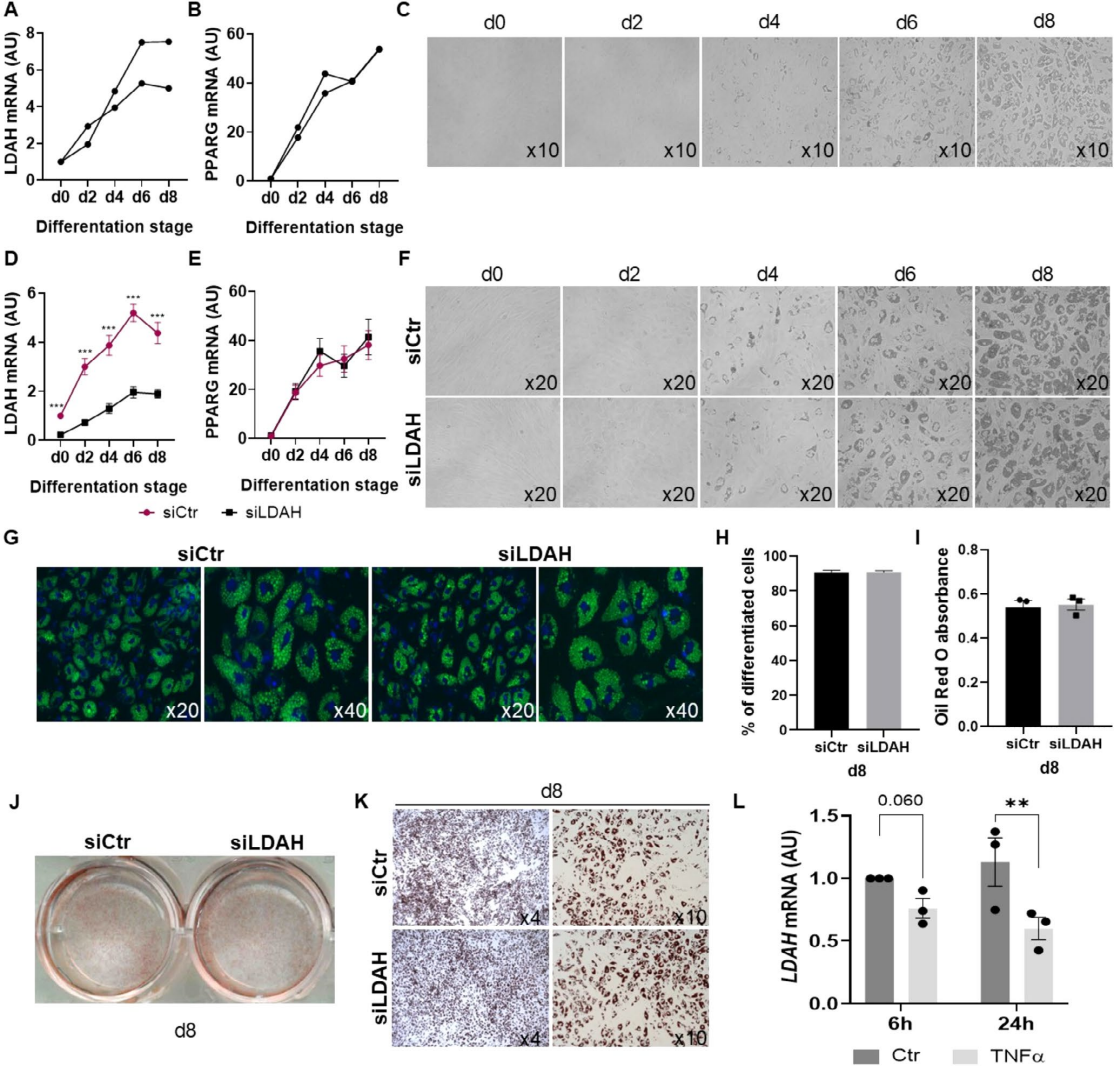
*LDAH* mRNA expression increases during the differentiation of SGBS cells but is not essential for human adipocyte differentiation. (A, B) *PPARG* and *LDAH* mRNA expression in SGBS cells during 8 days of differentiation, respectively, in two independent experiments. Results are shown relative to Day 0 (set to 1). (C) Representative bright‐field images of SGBS cell differentiation into mature adipocytes. (D, E) *PPARG* and *LDAH* mRNA expression, respectively, in *LDAH* knockdown (siLDAH) SGBS cells and SGBS control cells (siCtr) during differentiation in five independent experiments. Results are shown relative to control Day 0 (set to 1). (F) Representative bright‐field images of siLDAH and siCtr SGBS cell differentiation into mature adipocytes. (G) Representative images of siCtr and siLDAH SGBS cells at Day 8 of differentiation documented by Nile Red/Hoechst double staining. (H) No differences in the percentage of differentiated SGBS cells at Day 8 in siCtr and siLDAH cells in three independent experiments. (I–K) No difference in SGBS adipocyte differentiation between siCtr and siLDAH cells, indicated by Oil Red O staining at Day 8, in a single representative experiment including Oil Red O absorbance measurement. (L) *LDAH* mRNA expression levels in differentiated SGBS adipocytes not treated (Ctr) or treated with TNFα (TNFα) for 6 and 24 h. Bar plots show SEM from three independent experiments. Results are shown relative to control 6 h (set to 1). AT, adipose tissue; d0, Day 0, immediately before induction of differentiation; d2, Day 2 of differentiation; d4, Day 4 of differentiation; d6, Day 6 of differentiation; d8, Day 8 of differentiation; LDAH, lipid droplet‐associated hydrolase; PPARG, peroxisome proliferator‐activated receptor gamma; siCtr, SGBS control cells for the *LDAH* knockdown; siLDAH, *LDAH* knockdown SGBS cells. Data in graphs expressed by mean and SEM. One‐way ANOVA with Holm‐Sidak post hoc method was used to compare expression during differentiation between siCtr and siLDAH cells. To compare the percentage of differentiated SGBS cells between siCtr and siLDAH cells, Student's *t*‐test was used. Mann–Whitney *U* test was applied to compare absorbances from Oil Red O staining between siCtr and siLDAH cells. **p < 0.01; ***p < 0.001. [Color figure can be viewed at wileyonlinelibrary.com]

As a first step to assess a potential direct role of LDAH in human adipocyte formation, *LDAH* gene expression was analyzed during adipocyte differentiation of human SGBS cells in vitro and compared to that of *PPARG*, the master regulator of adipogenesis. During the differentiation of SGBS preadipocytes into adipocytes, *LDAH* expression increased in a similar pattern to *PPARG*, although to a lesser extent. At Day 8 of adipocyte differentiation, the expression of *LDAH* was approximately 6‐fold higher compared to Day 0 (Figure 3A), while *PPARG* expression was increased to about 50‐fold (Figure 3B), confirming successful and efficient adipocyte differentiation, as also indicated by the content of lipid droplets increasing with time (Figure 3C).

### 3.5 Adipocyte Differentiation Does Not Seem to Be Affected by *LDAH* Knockdown

To investigate whether *LDAH* expression might be essential for adipogenesis, knockdown of *LDAH* in SGBS preadipocytes was performed using gene‐specific siRNA. Efficient knockdown of *LDAH* to 75% at d0 of adipocyte differentiation was confirmed using qPCR (Figure 3D). Knockdown efficiency was maintained above 50% throughout the adipocyte differentiation period, with *LDAH* mRNA expression being 55.64% ± 0.04% less in knockdown cells (siLDAH) compared to control cells (siCtr) at Day 8 of adipocyte differentiation.

Knockdown of *LDAH* did not affect differentiation of SGBS cells into adipocytes as indicated by no significant changes in mRNA expression of *PPARG* (Figure 3E) and no visual differences in number or morphology of lipid droplets between siLDAH and siCtr cells (Figure 3F). An analysis of differentiation capacity by Nile Red/Hoechst staining (Figure 3G) showed the same rates in both cell types at Day 8 (90.57% ± 2.88% differentiated adipocytes in siCtr cells vs. 90.62% ± 1.66% in siLDAH cells, *p* = 0.976; Figure 3H). Likewise, Oil Red O staining at Day 8 did not show significant differences in lipid accumulation between siCtr and siLDAH (Figure 3I–K). Taken together, LDAH does not seem to be involved in adipocyte differentiation in vitro.

### 3.6 TNFα Decreases *LDAH* mRNA Expression in Vitro

To confirm the observed association between *LDAH* expression and AT inflammation, we finally performed in vitro analyses and determined *LDAH* mRNA expression levels in differentiated SGBS adipocytes treated with the proinflammatory cytokine TNFα. Cells treated with TNFα for 6 h showed a trend towards lower expression of *LDAH* compared to control cells without TNFα treatment, which was, however, not significant, while cells treated with TNFα for 24 h showed significantly lower levels of *LDAH* mRNA expression compared to control cells (*p* = 0.009, Figure 3L). These results point towards a possible negative regulation of the expression of *LDAH* by TNFα and align with the results found in AT samples of children, reinforcing the idea of a negative association of *LDAH* with AT inflammation.

## 4 Discussion

Due to its association with lipid droplets and its lipase‐like structure, we hypothesized that LDAH might play a potential role in AT and might be associated with obesity and related metabolic alterations. Here, we analyzed *LDAH* mRNA expression in subcutaneous AT from children included in the Leipzig Adipose Tissue Childhood Cohort, as well as during adipocyte differentiation in vitro. While we did not find evidence for a direct effect of LDAH on adipocyte differentiation in SGBS cells, we observed an association of AT *LDAH* mRNA expression with obesity and AT inflammation in children.

While some previously published studies have analyzed the role of LDAH in lipid mobilization in *D. melanogaster* , *S. cerevisiae* , HEK293 cells, and mouse macrophages [13, 16, 18, 36, 37, 38], there is a lack of studies that explore a potential direct role in human AT and an association with obesity and related disease. We first explored a potential association of genetic variation in *LDAH* with obesity by analyzing a specific SNP (rs13385191) within the *LDAH* gene. The selection of this SNP was motivated by its established association with *LDAH* gene expression in prostate and liver in several studies [22, 25, 27], as well as its reported link to disease conditions, in particular prostate cancer [20, 21, 22, 24, 25, 26, 27]. We hypothesized that the same genetic variant might influence *LDAH* expression and its metabolic implications in AT. Genotyping results analysis revealed that carriers of the G allele are linked to reduced *LDAH* expression in AT, which aligns with previous studies [14, 22, 25, 27]. In our cohort, this allele was also associated with higher BMI, which is consistent with reports showing that genetic variations in the *LDAH* locus are linked to lipid‐related traits and anthropometric measures [14], suggesting broader cardiometabolic relevance beyond cancer.

We next characterized a potential association of *LDAH* mRNA expression in AT with obesity and AT biology in children. The lack of significant correlations with height, sex, or pubertal stage in healthy lean children suggests that AT *LDAH* expression is not directly affected by normal growth or sexual development of children. Also, the observed negative correlation between AT *LDAH* expression and age is rather weak, and its physiological relevance needs to be addressed in future studies.

When the analysis was extended to the entire cohort including lean children and children with overweight and obesity, we observed a negative correlation with BMI SDS, indicating that lower *LDAH* expression is associated with higher BMI. These findings are in line with the notion that LDAH may be linked to metabolic health, suggesting a potential role for LDAH in adiposity, as these correlations remained significant even after adjusting for sex and age/puberty stage as covariates. Importantly, this is consistent with the results found by Currall et al., which demonstrated that the loss of LDAH in mice was related to increased body weight in females [14].

Furthermore, we observed a significant negative correlation between AT *LDAH* expression and AT inflammation as a parameter of obesity‐related AT dysfunction. This pattern was supported by concordant readouts obtained using independent approaches, such as macrophage quantification, CLS assessment, macrophage marker gene expression, and the association with lower circulating TNFα levels in children with higher *LDAH* expression. Together, these findings might point to a potential protective role of LDAH in mitigating AT inflammation and dysfunction. Our findings align with previous studies demonstrating that LDAH promotes less inflammatory macrophage phenotypes by mobilizing bioactive lipids, including natural ligands of the liver X receptor (LXR), and is associated with a reparative/profibrotic signature in atherosclerosis models [18]. This mechanism might extend to AT, where macrophages significantly contribute to local inflammation and CLS formation [39]. Notably, although LDAH has been reported to modulate ATGL‐dependent lipid mobilization in other cellular systems [15, 17], we did not observe an association between *LDAH* expression and either basal or isoproterenol‐stimulated lipolysis in our pediatric AT samples, nor did we observe an association with adipocyte diameter, arguing against a major contribution of adipocyte lipolysis or hypertrophy to the observed inflammatory associations in this setting. Overall, our data support a link between *LDAH* expression and adipose immune remodeling in children, in line with a broader role of lipid‐droplet biology in shaping tissue inflammation.

The link between *LDAH* expression and inflammation is further supported by the mechanistic in vitro analyses in SGBS preadipocytes performed in this study. In light of its association with childhood obesity, we first characterized *LDAH* mRNA expression during differentiation of SGBS preadipocytes into adipocytes, since no study had assessed it in human adipocytes to date, and found a steady increase with ongoing adipocyte differentiation (Figure 3A). This agrees with results previously found in mouse 3 T3‐L1 adipocytes [15]. Therefore, we hypothesized that LDAH may be directly involved in adipocyte differentiation, but we failed to find any difference between *LDAH* knock down and control cells. Consistently, we did not observe increased lipid accumulation or larger lipid droplets upon *LDAH* silencing, which would be expected if LDAH markedly promoted net lipid storage by limiting lipid mobilization under our conditions. Although previous studies in HEK293 cells suggested that LDAH may promote tubulin‐dependent lipid droplet fusion [15], we did not detect clear lipid droplet morphological changes; however, more precise methods should be used to validate this. Moreover, although we achieved a robust and stable knockdown, we cannot exclude that the residual amount of LDAH is sufficient for adipocyte differentiation or that other lipid‐associated hydrolases partially compensate for loss of LDAH.

Importantly, our in vitro results further reinforce the connection of AT *LDAH* expression to inflammation. Treatment of human adipocytes with TNFα decreased *LDAH* expression, indicating that the negative relationship between AT inflammation and *LDAH* expression may be partially mediated by a direct effect of inflammatory factors on *LDAH* expression.

While our results align with existing literature, our study is limited by the lack of direct mechanistic data in AT. Unfortunately, we were limited by the often‐small amount of AT obtained from children during elective surgeries, which precluded quantification of LDAH protein levels and additional mechanistic analyses, as well as functional validation or the consideration of depot‐specific effects. Also, the observed correlations with AT inflammation are relatively weak, though consistent through multiple independent readouts and complemented with results from in vitro studies. Future clinical and mechanistic research should aim to elucidate the pathways through which LDAH influences AT health, focusing on its physiological and clinical relevance in the context of childhood obesity.

In summary, our study provides new insights into the role of LDAH in adiposity and inflammation showing that higher *LDAH* expression is associated with a healthier adipose microenvironment, with significant implications for metabolic diseases, including childhood obesity. These findings bridge molecular mechanisms and metabolic phenotypes, laying the groundwork for future investigations into LDAH and its role in metabolic health, with potential future applications in early diagnosis, risk stratification, and therapeutic development for childhood obesity and related diseases.

## Funding

This work was supported by the Deutsche Forschungsgemeinschaft (DFG, German Research Foundation)—Projektnummer 209933838—SFB 1052, by the Federal Ministry of Education and Research (BMBF) as part of the German Center for Child and Adolescent Health (DZKJ, funding code 01GL2405A), by funds from the German Diabetes Association (DDG) to AK, and by the German Diabetes Foundation (Deutsche Diabetes Stiftung, DDS; FP‐0464‐2025) and the Roland Ernst Foundation (project number 01/25) to KL. CV was the recipient of the M‐AES mobility grant, MV22/00010, by the Carlos III Institute of Health (ISCIII, Spain).

## Conflicts of Interest

The authors declare no conflicts of interest.

## Data Availability

The data that support the findings of this study are available from the corresponding author upon reasonable request.
